# Investigation of the Frequency and Severity of Sexual Dysfunctions in Patients with Alcohol, Drug and Gambling Addiction

**DOI:** 10.1192/j.eurpsy.2026.10840

**Published:** 2026-07-31

**Authors:** S. Oktar, G. Gulec

**Affiliations:** Psychiatry, Eskişehir Osmangazi University, Eskisehir, Türkiye

## Abstract

**Introduction:**

Addictive disorders may lead to various physical and psychological consequences, and one of the most frequently reported problems is sexual dysfunction (SD). SD can negatively affect treatment adherence, interpersonal relationships, and overall quality of life. Although the relationship between alcohol or substance addiction and SD has been investigated in several studies, comparative studies including gambling disorder (GD) remain limited. Considering the shared reward system mechanisms among these addictions, exploring sexual functioning across different addiction types may provide valuable clinical insights.

**Objectives:**

To compare the **frequency and severity of sexual dysfunctions** among individuals with **alcohol use disorder (AUD)**, **substance use disorder (SUD)**, and **gambling disorder (GD)**, and to assess their differences from a matched healthy control group.

**Methods:**

The study included 174 patients: 54 with GD, 60 with AUD, and 60 with SUD, recruited from the AMATEM Unit of Eskişehir Osmangazi University Faculty of Medicine and the AMATEM Service of Eskişehir Yunus Emre State Hospital. Sixty healthy volunteers matched for age, gender, and education served as the control group. Participants completed the Sexual Myths Scale (SMS), Sexual Sensation Seeking Scale (SSSS), and Golombok–Rust Sexual Satisfaction Scale (GRISS). The South Oaks Gambling Screen (SOGS) was administered to the GD group, the Severity of Alcohol Dependence Questionnaire (SADQ) and the Addiction Profile Index (BAPI) to the AUD group, and the BAPI to the SUD group.

**Results:**

Sexual dysfunction was found in 57.4% of the GD group, 76.6% of the AUD group, 78.3% of the SUD group, and 70% of controls. Compared with controls, the addiction group had higher SMS total and subscale scores except for two subscales. No significant differences were found in SSSS or GRISS total scores. Among addiction subgroups, SSSS scores did not differ, while the GRISS premature ejaculation subscale was higher in the AUD and SUD groups than in the GD group. SMS total and subscale scores were higher in six subscales for SUD, two for AUD, and one for GD compared with controls.

**Conclusions:**

Sexual dysfunctions were common in all addiction types, particularly among individuals with alcohol and substance use disorders. Findings suggest that sexual problems may be linked to distorted sexual beliefs and to physiological and psychological effects of substance use rather than increased sexual drive. Addressing sexual functioning and myths during treatment may enhance recovery and relationship satisfaction. Further studies with larger samples and longitudinal designs are needed to clarify causal mechanisms.

**Keywords:**

Addiction, substance use disorder, gambling disorder, sexual dysfunction

**Disclosure of Interest:**

None Declared

